# Preimplantation genetic testing for Aicardi–Goutières syndrome induced by novel compound heterozygous mutations of *TREX1*: an unaffected live birth

**DOI:** 10.1186/s13039-023-00641-5

**Published:** 2023-06-05

**Authors:** Huiling Xu, Jiajie Pu, Suiling Lin, Rui Hu, Jilong Yao, Xuemei Li

**Affiliations:** 1 Department of Reproductive Medicine, Southern Medical University Affiliated Shenzhen Maternity and Child Healthcare Hospital, Shenzhen, Guangdong China; 2 Department of Bioinformatics, 01life Institute, Shenzhen, 518000 Guangdong China

**Keywords:** Aicardi–Goutières syndrome, *TREX1*, PGT-M, Monogenic disease

## Abstract

**Background:**

Aicardi–Goutières syndrome (AGS) is a rare, autosomal recessive, hereditary neurodegenerative disorder. It is characterized mainly by early onset progressive encephalopathy, concomitant with an increase in interferon-α levels in the cerebrospinal fluid. Preimplantation genetic testing (PGT) is a procedure that could be used to choose unaffected embryos for transfer after analysis of biopsied cells, which prevents at-risk couples from facing the risk of pregnancy termination.

**Methods:**

Trio-based whole exome sequencing, karyotyping and chromosomal microarray analysis were used to determine the pathogenic mutations for the family. To block the inheritance of the disease, multiple annealing and looping-based amplification cycles was used for whole genome amplification of the biopsied trophectoderm cells. Sanger sequencing and next-generation sequencing (NGS)-based single nucleotide polymorphism (SNP) haplotyping were used to detect the state of the gene mutations. Copy number variation (CNV) analysis was also carried out to prevent embryonic chromosomal abnormalities. Prenatal diagnosis was preformed to verify the PGT outcomes.

**Results:**

A novel compound heterozygous mutation in *TREX1* gene was found in the proband causing AGS. A total of 3 blastocysts formed after intracytoplasmic sperm injection were biopsied. After genetic analyses, an embryo harbored a heterozygous mutation in *TREX1* and without CNV was transferred. A healthy baby was born at 38th weeks and prenatal diagnosis results confirmed the accuracy of PGT.

**Conclusions:**

In this study, we identified two novel pathogenic mutations in *TREX1*, which has not been previously reported. Our study extends the mutation spectrum of *TREX1* gene and contributes to the molecular diagnosis as well as genetic counseling for AGS. Our results demonstrated that combining NGS-based SNP haplotyping for PGT-M with invasive prenatal diagnosis is an effective approach to block the transmission of AGS and could be applied to prevent other monogenic diseases.

**Supplementary Information:**

The online version contains supplementary material available at 10.1186/s13039-023-00641-5.

## Background

Aicardi–Goutières syndrome (AGS) (OMIM 225750) was first described in 1984 by Jean Aicardi and Francoise Goutières [1]. It has a prevalence of 1–5 in 10,000 newly live births [2]. AGS is an autoimmune and genetically heterogeneous disorder with severe neurologic injury [3]. Affected patients’ onset usually occurs within the first year of life, and around 25% of them die between the age of 1 and 17 years [4]. AGS primarily affects the brain and the skin. It is most frequently inherited in an autosomal recessive manner and seems to exhibit high variations in penetrance even among patients with same variant [4, 5]. The main clinical features include encephalopathy, leukodystrophy, cerebral atrophy, multiple intracranial calcifications, and lymphocytosis and raised levels of interferon-alpha (IFNα) in the cerebrospinal fluid (CSF) [6]. Nine pathogenic genes associated with AGS have been identified, including *TREX1*, *RNASEH2B*, *RNASEH2C*, *RNASEH2A*, *SAMHDl*, *LSM11*, *RNU7-1*, *ADAR1* and *IFIH1* [7, 8].

TREX1 is a 314 amino-acid-long protein encoded by a single exon which is located at 3p21. When preparing this manuscript, there are 197 *TREX1* mutations have been recorded in ClinVar database (https://www.ncbi.nlm.nih.gov/clinvar/). TREX1 is a potent 3’–5’ exonuclease that degrades single and double-stranded DNA. When TREX1 mutates, it will fail to appropriately disassemble genomic DNA during normal cell death processes, which could trigger the innate immune response and autoimmunity [9, 10]. Unfortunately, the main treatment for AGS is only symptomatic. There are still a number of issues regarding AGS treatment needed to be addressed [11, 12].

PGT is a procedure used to analyze gametes or embryos, then unaffected embryos are transferred back to women’s uterus, which allow at-risk couples having unaffected children of their own without facing the risk of pregnancy termination [13, 14]. The PGT includes three sub-categories: PGT for aneuploidies (PGT-A), PGT for single monogenic disorders (PGT-M), and PGT for chromosome structural rearrangements (PGT-SR) [15]. In this study, we report a clinically diagnosed AGS caused by a novel compound heterozygous mutation in *TREX1* gene in a Chinese family. These two variants have not been reported previously and have not been recorded as a polymorphic change in public databases [ClinVar (https://www.ncbi.nlm.nih.gov/clinvar), HGMD (http://www.hgmd.org), LOVD (http://www.lovd.nl)]. To prevent the subsequent transmission of pathogenic mutations in next generation, the couple was counselled and suggested to receive PGT-M treatment. The whole process includes ovarian stimulation, oocyte retrieval, in vitro fertilization, embryo culture and biopsy, and frozen embryo transfer. Through precise detection procedures, an unaffected embryo was transferred, and a healthy female baby was born at full term.

## Methods

### Case description

In this case, the Han Chinese none-consanguineous couple were healthy. The mother had her pregnancy at the age of 30. A detailed ultrasound scan at 30 weeks of gestation showed microcephaly and multiple intracranial calcifications. To identify the potential genetic variants through Trio-WES, total genomic DNA was extracted from the whole blood of the couple and amniocytes of the proband. DNA fragments were hybridized and captured by the ClearSeq Inherited Disease Panel (Agilent Technologies, Inc, USA) according to manufacturer's protocol. Then the sequencing library was sequenced on the NovaSeq 6000 platform (Illumina, Inc, USA) with 150 bp paired-end reads. The sequencing reads were aligned to the human reference genome (hg19/GRCh37) with BWA (v0.7.17). Conventional G-banded karyotyping test and CMA test with CytoScan 750 K (Affymetrix, Inc, USA) arrays were performed according to the manufacturer's manual to detect chromosome abnormalities.

### In vitro fertilization and blastomere biopsy

The ovarian stimulation, in vitro fertilization, embryo culture and transfer processes were conducted according to the routing protocols [16–18]. In our case, 12 oocytes were obtained and then inseminated by ICSI. Five of them were fertilized normally as indicated by the presence of two pronuclei. In total, 3 blastocysts were obtained. All of them were subjected to TE biopsy on day 5 via laser. Approximately 5–10 cells were extracted from each blastocyst and ready for WGA. The IVF and embryo biopsy were performed at the Center of Reproductive Medicine of the Shenzhen Maternity and Child Healthcare Hospital. WGA and NGS-based PGT were done at Yikon Genomics (Suzhou, China).

### PGT with haplotype analysis

Biopsied TE cells from each embryo were applied following the standard protocol of WGA method of MALBAC (Yikon Genomics) [19]. The WGA products of each embryo were subjected to Sanger sequencing to identify mutation directly. Because only a few cells are available for amplification, it is hard to avoid allele drop-out (ADO) and is related to the increased risk of DNA contamination. To prevent misdiagnosis, haplotype analysis was conducted through SNP markers. During the pre-testing, SNPs within the 2 Mb region flanking *TREX1* gene are genotyped via NGS using DNA samples from the couple and their parents since the proband’s DNA sample was degraded. Informative SNPs that allow discrimination of the high-risk haplotype and low-risk haplotype are selected for use in the embryo test. A total of thirty SNPs were chosen and screened to determine the possible embryo genotypes via targeted capture sequencing (TCS) with sequencing depth ≥ 100× , and specific primers were designed online (http://www.ampliseq.com).

### CNV analysis

Besides the detection of *TREX1* mutation, CNV analysis was also carried out using WGA products to prevent embryonic abortion, death or other problems may be caused by embryonic chromosomal abnormalities. Any deletions or duplications more than 4Mbp and mosaicism more than 30% in each embryo were reported. Rrefer to previous study for details [20].

### Embryo transfer and prenatal diagnosis

The selection for embryo transfer was based on the morphology and the PGT results. Clinical pregnancy was defined as the presence of a fetal heartbeat by sonography 35 days after frozen embryo transfer (FET). To verify the PGT diagnosis, Sanger sequencing, karyotyping and CMA were conducted using the fetal DNA obtained through amniocentesis at the 17 weeks of gestation.

## Results

### Genetic analysis

**Fig. 1:**
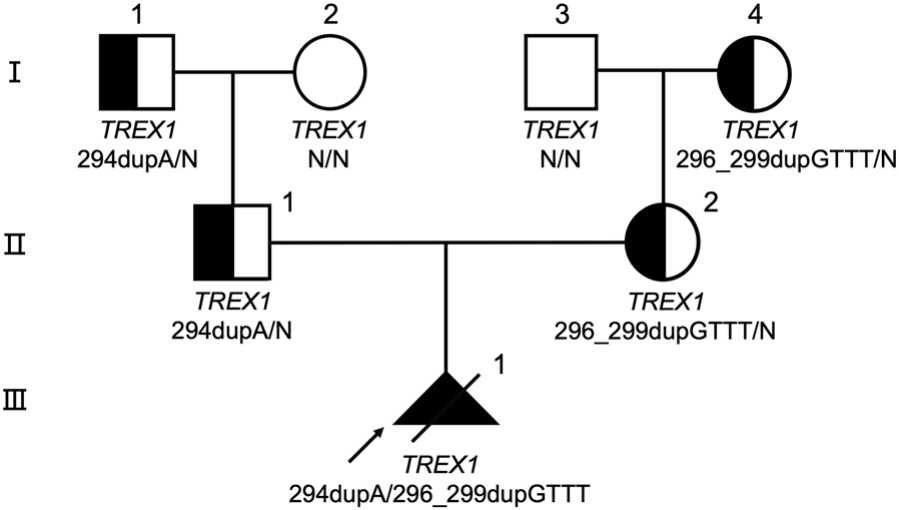
Pedigree of the AGS family reported in this study. Squares and circles indicate males and females, respectively. The arrowhead denotes the proband

As the karyotyping and CMA results showed that the proband had a normal 46, XY karyotype without any deletions or duplications. Trio-WES revealed that the proband harbored a compound heterozygous mutation in *TREX1* gene (maternal: c.296_299dupGTTT, p.Phe100LeufsTer3; paternal: c.294dupA, p.Cys99MetfsTer3) (Fig. 1), which is confirmed by Sanger sequencing. These two mutations were classified as likely pathogenic mutation according to the American College of Medical Genetics and Genomics (ACMG) guideline (PVS1, PM2, PP3) (Additional file 1: Table S1) [21]. Both mutations were predicted to introduce a premature stop codon that affect TREX1 protein function. Mutation c.294dupA caused the TREX1 peptide chain shortened from 314 to 202 amino acids. The other mutation c.296_299dupGTTT led to premature translational termination at amino acid position 222, thus the peptide chain was shortened from 314 to 221 amino acids. Pregnancy termination was requested by the parents at the 34 weeks of gestation. Then the couple at risk were underwent PGT treatment.

### Embryo preparation and genetic testing

**Fig. 2:**
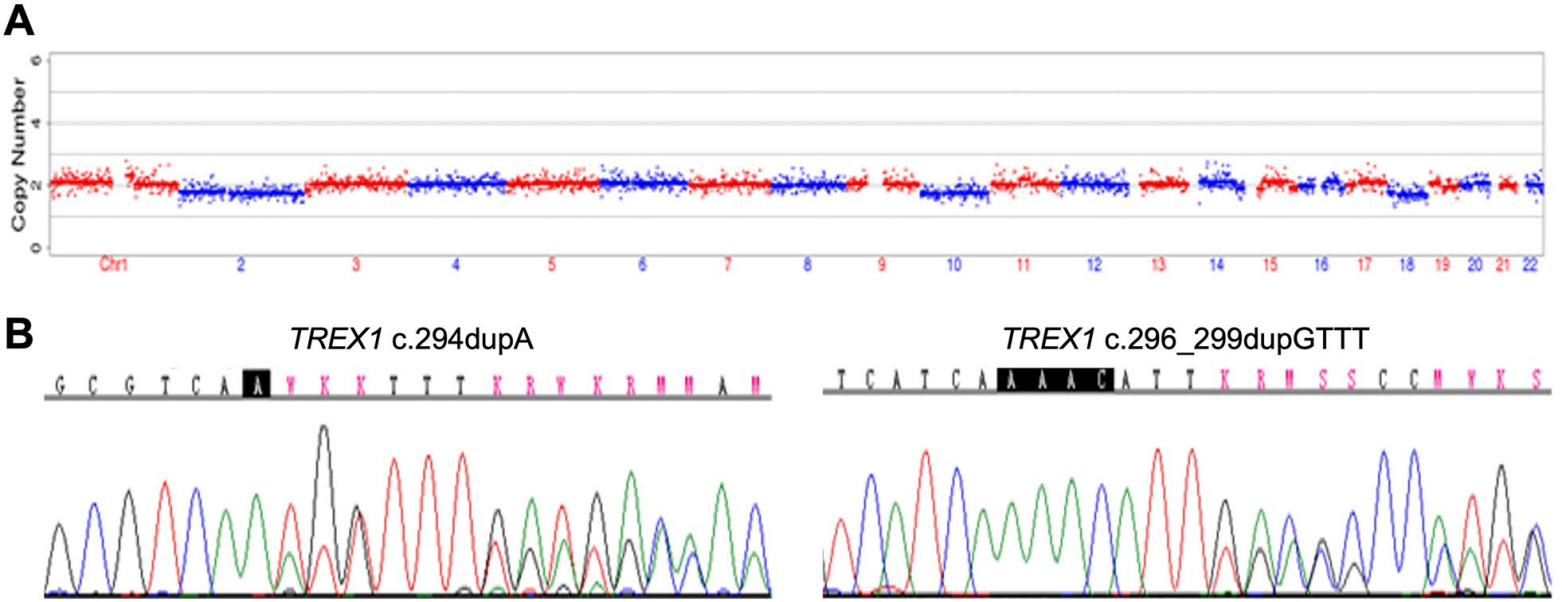
Copy number variations and Sanger sequencing results of the embryo E2.** A** The sketch map showed that E2 was euploidy.** B** Sanger sequencing result showed that E2 carried the *TREX1* mutation c.294dupA

**Fig. 3:**
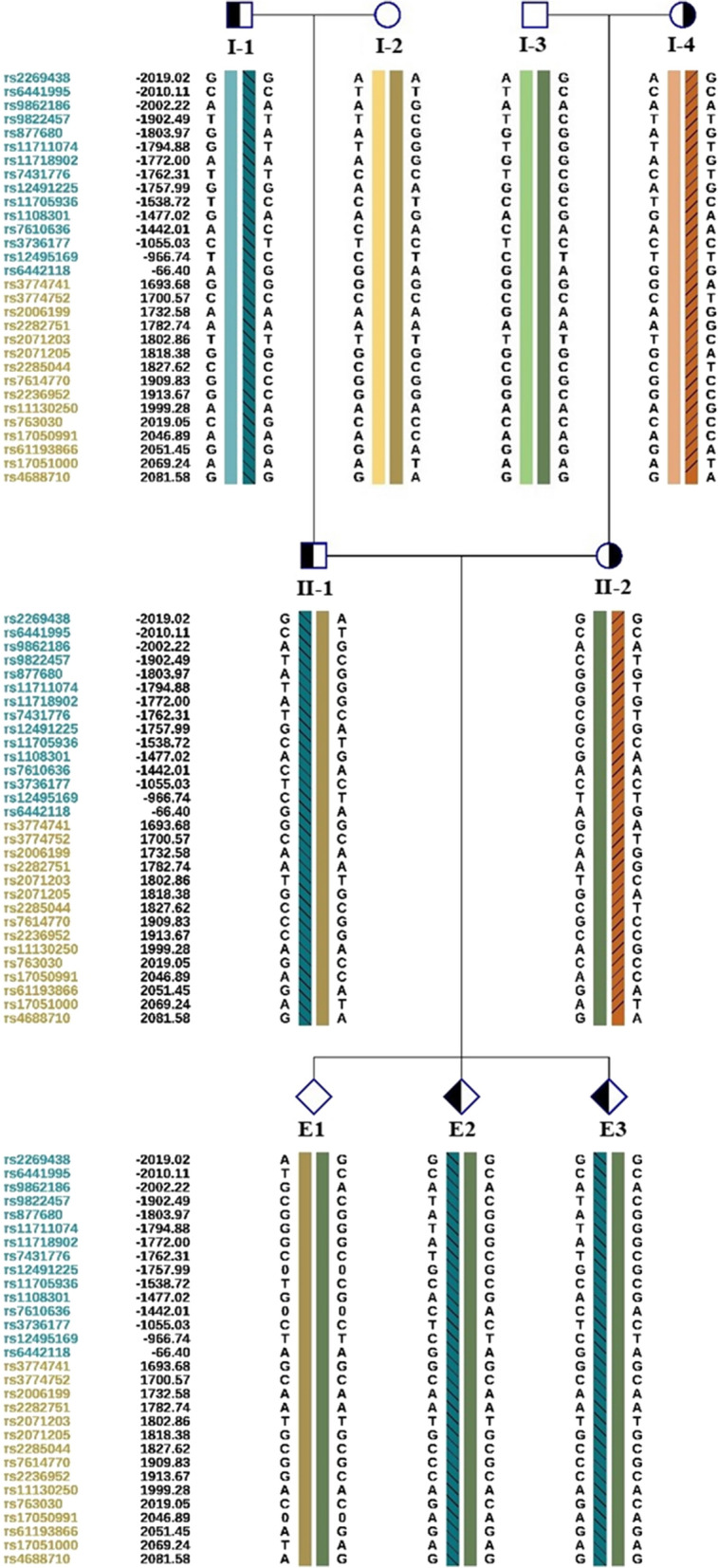
Pedigree of haplotype linkage analysis. The backslash represents a carrying haplotype inherited from the I-1 and II-1, whereas slash represents a carrying haplotype inherited from I-4 and II-2. Colored rectangle without slash or backslash represents a normal haplotype

Among the five embryos fertilized by ICSI, three of them (E1, E2, E3) were suitable for biopsy (Table 1). We successfully amplified the DNA of the blastocyst by MALBAC. The CNV results (Fig. 2A, Additional file 3: Fig. S1) showed that only E2 was diagnosed as normal karyotype. Other two embryos showed chromosomal abnormalities related to varying degrees of chromosome mosaicism. After Sanger sequencing (Fig. 2B, Additional file 3: Fig. S1), no mutation was found in *TREX1* for E1, but both E2 and E3 carried the mutation c.294deupA. To verify the Sanger sequencing results, we selected 30 SNPs that could be used for determination of the high-risk haplotype and the low-risk haplotype after pre-testing to infer the disease status of the embryos (Table Additional file 2: S2 and Fig. 3). SNP-based haplotyping showed that both E2 and E3 inherited the paternal high-risk chromosome carrying c.294dupA mutation, while E1 carried neither mutation, which was consistent with the Sanger sequencing.

**Table 1:** Detection result summary of the three biopsied blastocysts

Embryo number	Grading#	Sanger sequencing	SNP haplotyping	CNV
E1	3BB	normal	Wild type haplotype	Mixed aneuploidy*
E2	3BB	c.294dupA	Paternal haplotype carrier	46,XN
E3	3CB	c.294dupA	Paternal haplotype carrier	46,XN,4p(pter → p12, ~ 49 Mb, × 1,mos, ~ 50%), 4q(q12 → q35.2, ~ 138 Mb, × 1,mos, ~ 50%)

^#^The embryo quality was assessed following the Gardner grading system [22]

^*^Embryo with both aneuploid and mosaic chromosomes

### Prenatal diagnosis and pregnancy outcome

Based on embryo selection principles, the euploid embryo E2 carried heterozygous mutation c.294dupA in *TREX1* was transferred into uterus. During the pregnancy, ultrasound scan showed that the fetus was well developed without microcephaly or intracranial calcifications. The prenatal diagnosis result was consistent with PGT-M. Finally, a healthy female infant was born at the 38th week of gestation.

## Discussion

Birth defects bring serious psychological and financial burden to families and have become a crucial social problem. It is estimated that genetic factors contribute solely or collaboratively to about 80% of the occurrence of birth defects. Therefore, genetic studies can provide precise molecular targets for clinical screening, diagnosis and treatment [23]. In this study, we detected two novel missense mutations in *TREX1* gene (c.294dupA and c.296_299dupGTTT) in an AGS fetus, which were inherited from the normal father and mother, separately. Through PGT-M, an embryo was confirmed as euploid as well as heterozygous carrier status of *TREX1* gene, which was verified by Sanger sequencing and haplotype analysis. After transferring the selected embryo, a health fetus was born.

PGT has developed by leaps and bounds since 1989s [24]. It prevents not only the invasive prenatal test which may constitute a risk to the fetus but also the abortion with bearing an affected fetus that may impose psychological and physical burdens on the pregnant woman. However, due to the limited DNA amount of biopsied cells, direct genetic testing through Sanger sequencing based on PCR has the risk of ADO, which is one of the most important causes of the misdiagnoses. SNPs are the most abundant form of human genetic variations, about one SNP every 100–300 bp [25]. And they are easy to interpret and amenable to high-throughput analysis. SNP-based haplotyping could be used to evaluate the occurrence of ADO, monosomy, trisomy and DNA contamination [26]. A clear discrimination between the high-risk and low-risk allele can be made with fully informative SNP markers located close to the target gene. Here, we used NGS-based SNP haplotyping for the verification of the Sanger sequencing results. Such highly informative haplotypes could improve the accuracy and reliability of embryo diagnosis.

Chromosomal mosaicism is at high prevalence in early developmental stage of embryos, which is much higher than reported in the prenatal and postnatal cytogenetic literature [27]. Nowadays, Chromosomal mosaicism is a potential factor of misdiagnosis and has been a raising problem in PGT [28]. A single trophectoderm biopsy of a few cells couldn’t represent the entire embryo. Besides, amplification bias in WGA, sample DNA biological variability, sequencing methods and bioinformatic approaches may lead to false positives. Thirdly, embryo mosaicism may self-correct aneuploidy downstream [29, 30]. Recent studies provided evidence that mosaic embryos can develop into healthy euploid newborns [31–33]. Compared with euploid embryo transfer, however, several studies have reported that mosaic embryo transfer is associated with a lower rate of live birth and a higher rate of biochemical miscarriage [34–36]. Considering the possible risks of transferring mosaic embryos, we eventually selected an euploid carrier embryo E2 to transfer. In this study, rate of mosaicism seems more frequent than reported for that two of the three embryos were mosaic. A possible explanation might be that the use of laser pulse in biopsy improperly increased cell damage which influenced the sequencing results, leading to misdiagnosis of a normal embryo as mosaic. Furthermore, embryo E1 is a complex mosaic blastocyst which involves more than three chromosomes. Recently, a retrospective study showed that 61.6% of the complex mosaic were diagnosed as euploidy in re-biopsy. And the prevalence of the complex mosaic was 2.4% [37]. Therefore, embryos exhibit complex mosaicism, like the E1 in our case, could be re-biopsied to avoid waste of potential available embryos.

We report here our experience of applying NGS-based SNP haplotyping for PGT-M for a couple carrying different mutations in *TREX1* which led to the pregnancy termination of an affected fetus with AGS. According to the age of onset, two clinical presentations of AGS could be delineated: early-onset neonatal form and later-onset presentation. The former highly reminiscent of congenital infection seen particularly with *TREX1* mutations [38]. In our study, 30-week ultrasound scan of the proband showed microcephaly and multiple intracranial calcifications which was initially misdiagnosed as acquired in utero viral infection. *TREX1* playing an important role in processing or clearing anomalous DNA structures, failure of which results in the triggering of an abnormal innate immune response, therefore AGS is sometimes mistaken as the sequelae of congenital infection [10]. Genetic testing is of obvious importance for distinguishing AGS from common perinatal infections.

In conclusion, our study found two novel mutations in *TREX1* gene in a family suffering from AGS. Our study combined NGS-based SNP haplotyping for PGT-M with invasive prenatal diagnosis could effectively block the subsequent transmission of *TREX1* genetic defects, resulting in the birth of a healthy baby. This approach was proven to be practicable while effective and could be expanded to other monogenic diseases.

## Supplementary Information

**Additional file 1**. Table S1. Variants classification following ACMG guidelines.

**Additional file 2**. Table S2. NGS-based SNP haplotyping results of the embryo E2.

**Additional file 3**. Fig S1. Copy number variations and Sanger sequencing results of the embryo E1 and E3.

## Data Availability

The datasets generated and/or analyzed during the current study are not publicly available due to individual privacy or ethical restrictions but are available from the corresponding author on reasonable request.
