# Supplementary material for: Preimplantation genetic testing for Aicardi–Goutières syndrome induced by novel compound heterozygous mutations of TREX1: an unaffected live birth
Source: Mol Cytogenet. 2023 Jun 5;16:9. doi: 10.1186/s13039-023-00641-5 (PMC10242808; doi:10.1186/s13039-023-00641-5)
Supplement: Supplementary file 1 — Additional file 1. Table S1. Variants classification following ACMG guidelines. [file 13039_2023_641_MOESM1_ESM.docx]

**Tab. S1 Variants classification following ACMG guidelines**

| **Variant** | **Evidence of pathogenicity** | | | | **Classification** |
| --- | --- | --- | --- | --- | --- |
|  | **Very strong** | **Strong** | **Moderate** | **Supporting** |  |
| c.296_299dupGTTT | PVS1 | Not applicable | PM2 | PP3 | likely pathogenic |
| c.294dupA | PVS1 | Not applicable | PM2 | PP3 | likely pathogenic |

PVS1: Null variant (frameshift) in a gene where loss of function (LOF) is a known mechanism of disease.

PM2: Absent from controls (or at extremely low frequency if recessive) in dbSNP, 1000 Genomes, Exome Variant Server or ExAC.

PP3: Multiple lines of computational evidence support a deleterious effect on the gene or gene product.

ExAC (http://exac.broadinstitute.org), dbSNP (<https://www>.ncbi.nlm.nih.gov/snp/), Exome Variant Server (<http://evs.gs.washington>.edu/EVS/), 1000 Genome Project ([www.1000genomes.org](http://www.1000genomes.org)), Polyphen-2 (<http://genetics.bwh.harvard.edu/pph2/)>, MutationTaster (https://www.mutationtaster.org/).
