# Supplementary material for: Preimplantation genetic testing for Aicardi–Goutières syndrome induced by novel compound heterozygous mutations of TREX1: an unaffected live birth
Source: Mol Cytogenet. 2023 Jun 5;16:9. doi: 10.1186/s13039-023-00641-5 (PMC10242808; doi:10.1186/s13039-023-00641-5)
Supplement: Supplementary file 2 — Additional file 2. Table S2. NGS-based SNP haplotyping results of the embryo E2. [file 13039_2023_641_MOESM2_ESM.docx]

**Tab. S2 NGS-based SNP haplotyping results of the embryo E2**

| **SNPs ID** | **I-1** | **I-2** | **II-1-normal** | **II-1-carry** | **I-3** | **I-4** | **II-2-normal** | **II-2-carry** | **E2- Paternal**  **haplotype** | **E2- Maternal**  **haplotype** |
| --- | --- | --- | --- | --- | --- | --- | --- | --- | --- | --- |
| **YK-SNP-1** | **G/G** | **A/A** | **A** | **G** | **A/G** | **A/G** | **G** | **G** | **G** | **G** |
| **YK-SNP-2** | **C/C** | **T/T** | **T** | **C** | **T/C** | **C/C** | **C** | **C** | **C** | **C** |
| **YK-SNP-3** | **A/A** | **A/G** | **G** | **A** | **A/A** | **A/A** | **A** | **A** | **A** | **A** |
| **YK-SNP-4** | **T/T** | **T/C** | **C** | **T** | **T/C** | **T/T** | **C** | **T** | **T** | **C** |
| **YK-SNP-5** | **G/A** | **A/G** | **G** | **A** | **G/G** | **A/G** | **G** | **G** | **A** | **G** |
| **YK-SNP-6** | **G/T** | **T/G** | **G** | **T** | **T/G** | **T/T** | **G** | **T** | **T** | **G** |
| **YK-SNP-7** | **A/A** | **A/G** | **G** | **A** | **G/G** | **A/G** | **G** | **G** | **A** | **G** |
| **YK-SNP-8** | **T/T** | **C/C** | **C** | **T** | **T/C** | **C/T** | **C** | **T** | **T** | **C** |
| **YK-SNP-9** | **G/G** | **A/A** | **A** | **G** | **G/G** | **A/G** | **G** | **G** | **G** | **G** |
| **YK-SNP-10** | **T/C** | **C/T** | **T** | **C** | **C/C** | **T/C** | **C** | **C** | **C** | **C** |
| **YK-SNP-11** | **G/A** | **AG** | **G** | **A** | **A/G** | **G/A** | **G** | **A** | **A** | **G** |
| **YK-SNP-12** | **A/C** | **C/A** | **A** | **C** | **C/A** | **A/A** | **A** | **A** | **C** | **A** |
| **YK-SNP-13** | **C/T** | **T/C** | **C** | **T** | **T/C** | **C/C** | **C** | **C** | **T** | **C** |
| **YK-SNP-14** | **T/C** | **C/T** | **T** | **C** | **C/T** | **T/T** | **T** | **T** | **C** | **T** |
| **YK-SNP-15** | **A/G** | **G/A** | **A** | **G** | **G/A** | **G/G** | **A** | **G** | **G** | **A** |
| **YK-SNP-16** | **G/G** | **G/G** | **G** | **G** | **G/G** | **G/A** | **G** | **A** | **G** | **G** |
| **YK-SNP-17** | **C/C** | **C/C** | **C** | **C** | **C/C** | **C/T** | **C** | **T** | **C** | **C** |
| **YK-SNP-18** | **A/A** | **A/A** | **A** | **A** | **G/A** | **A/G** | **A** | **G** | **A** | **A** |
| **YK-SNP-19** | **A/A** | **A/A** | **A** | **A** | **A/A** | **A/G** | **A** | **G** | **A** | **A** |
| **YK-SNP-20** | **T/T** | **T/T** | **T** | **T** | **T/T** | **T/C** | **T** | **C** | **T** | **T** |
| **YK-SNP-21** | **G/G** | **G/G** | **G** | **G** | **G/G** | **G/A** | **G** | **A** | **G** | **G** |
| **YK-SNP-22** | **C/C** | **C/C** | **G** | **C** | **C/C** | **C/T** | **C** | **T** | **C** | **C** |
| **YK-SNP-23** | **G/C** | **G/G** | **G** | **C** | **G/G** | **G/C** | **G** | **C** | **C** | **G** |
| **YK-SNP-24** | **G/C** | **G/G** | **G** | **C** | **G/C** | **G/C** | **C** | **C** | **C** | **C** |
| **YK-SNP-25** | **A/A** | **A/A** | **A** | **A** | **A/A** | **A/G** | **A** | **G** | **A** | **A** |
| **YK-SNP-26** | **C/G** | **C/C** | **C** | **G** | **C/C** | **A/C** | **C** | **C** | **G** | **C** |
| **YK-SNP-27** | **A/A** | **A/C** | **C** | **A** | **A/A** | **A/C** | **A** | **C** | **A** | **A** |
| **YK-SNP-28** | **G/G** | **G/A** | **A** | **G** | **G/G** | **G/A** | **G** | **A** | **G** | **G** |
| **YK-SNP-29** | **A/A** | **A/T** | **T** | **A** | **A/A** | **A/T** | **A** | **T** | **A** | **A** |
| **YK-SNP-30** | **G/G** | **G/A** | **A** | **G** | **G/G** | **G/A** | **G** | **A** | **G** | **G** |
