# Supplementary figures and images for: Preimplantation genetic testing for Aicardi–Goutières syndrome induced by novel compound heterozygous mutations of TREX1: an unaffected live birth
Source: Mol Cytogenet. 2023 Jun 5;16:9. doi: 10.1186/s13039-023-00641-5 (PMC10242808; doi:10.1186/s13039-023-00641-5)

**E1**

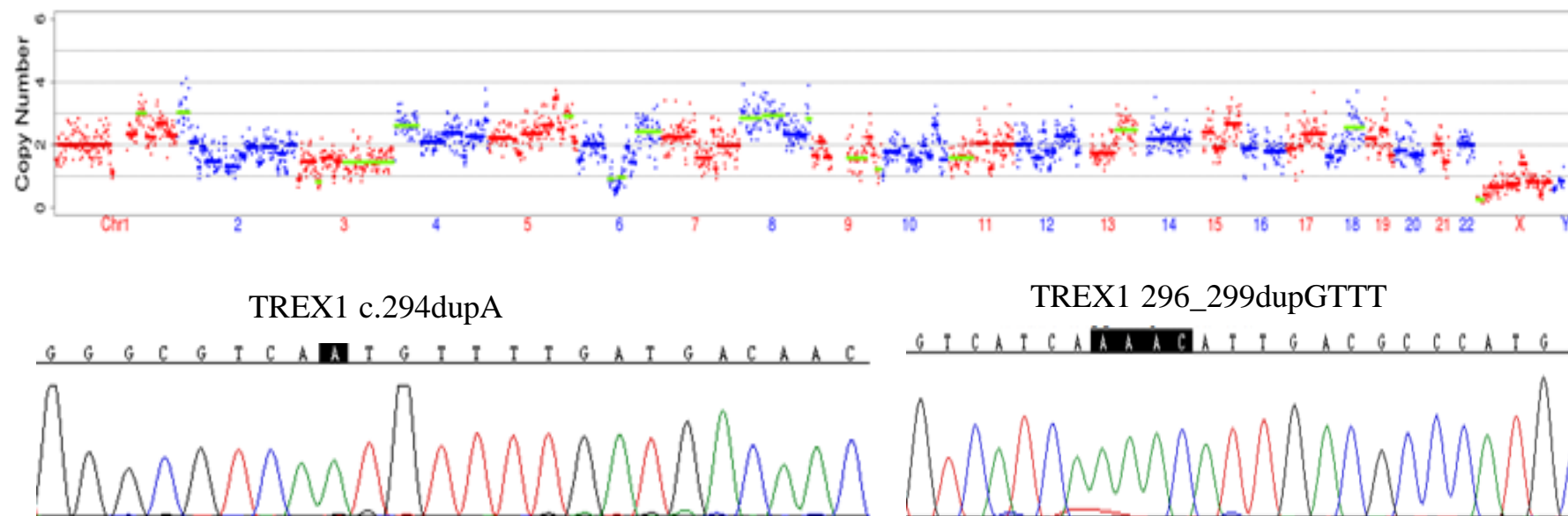

**E3**

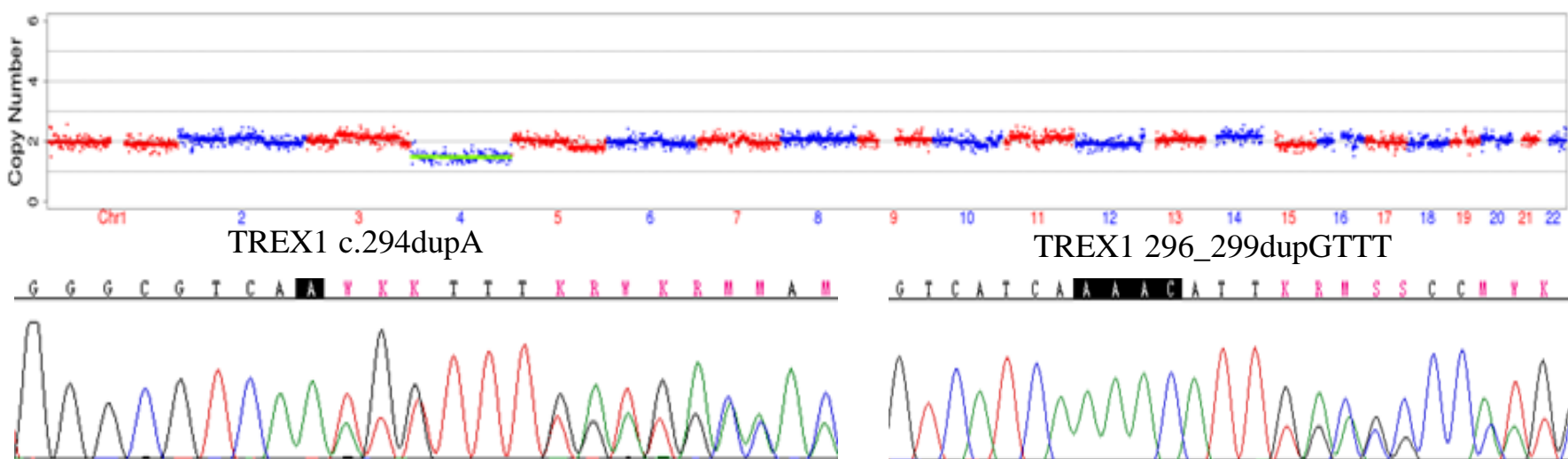

Supplement: Supplementary file 3 — Additional file 3. Fig S1. Copy number variations and Sanger sequencing results of the embryo E1 and E3. [file 13039_2023_641_MOESM3_ESM.pdf]
